# Wireless Sensor Network Localization via Matrix Completion Based on Bregman Divergence

**DOI:** 10.3390/s18092974

**Published:** 2018-09-06

**Authors:** Chunsheng Liu, Hong Shan, Bin Wang

**Affiliations:** Electronic Engineering Institute, National University of Defense Technology, Hefei 230037, China; HongShanWN@163.com (H.S.); BinWang806@163.com (B.W.)

**Keywords:** localization, matrix completion, Bregman divergence, pulse noise

## Abstract

One of the main challenges faced by wireless sensor network (WSN) localization is the positioning accuracy of the WSN node. The existing algorithms are arduous to use for dealing with the pulse noise that is universal and ineluctable in practical considerations, resulting in lower positioning accuracy. Aimed at this problem and introducing Bregman divergence, we propose in this paper a novel WSN localization algorithm via matrix completion (LBDMC). Based on the natural low-rank character of the Euclidean Distance Matrix (EDM), the problem of EDM recovery is formulated as an issue of matrix completion in a noisy environment. A regularized matrix completion model is established, smoothing the pulse noise by leveraging $L_{1,2}$-norm and the multivariate function Bregman divergence is defined to solve the model to obtain the EDM estimator. Furthermore, node localization is available based on the multi-dimensional scaling (MDS) method. Multi-faceted comparison experiments with existing algorithms, under a variety of noise conditions, demonstrate the superiority of LBDMC to other algorithms regarding positioning accuracy and robustness, while ensuring high efficiency. Notably, the mean localization error of LBDMC is about ten times smaller than that of other algorithms when the sampling rate reaches a certain level, such as >30%.

## 1. Introduction

Wireless sensor networks (WSNs) are widely used in monitoring, target tracking, and other fields [1,2], with the premise of providing accurate location information. Due to resource constraints, only a few beacon nodes in a WSN can implement their positioning by configuring GPS devices. In this case, the location information of unknown nodes can be achieved, employing the prior position coordinates of the beacon nodes as well as the physical measurements between the node pairs. In terms of the existing two kinds of WSN localization technologies [3], one uses range-based localization technology, which obtains distance or angle information depending on different ranging schemes (such as received signal strength (RSS) or time of arrival (TOA)). The other uses range-free localization technology, in which coarse-grained location information is acquired by using the connectivity between unknown nodes and beacon nodes [4].

As a crucial part of WSN applications, the localization problem in WSNs is of particular interest to researchers. The positioning accuracy is one of the main challenges in WSNs. Localization methods based on multi-dimensional scaling (MDS) [5,6,7], maximum likelihood (ML) [8], fingerprint [9,10], and semi-definite programming (SDP) [11] have been proposed.

The essence of the MDS-based localization method is in the nodes’ relative coordinates that are generated by the Euclidean Distance Matrix (EDM) being mapped to the absolute coordinates of the nodes, by aligning the coordinates of the beacon nodes [6]. However, the MDS method requires high precision of the EDM. In research by Bhaskar [8], the problem of node localization was described as a probabilistic problem and an algorithm based on the constrained maximum likelihood estimation was proposed in order to reconstruct the node position in *d*-dimensional Euclidean space. In addition, the relationship between the temporal correlation of RSS and positioning accuracy was studied in Wang’s research [9] and the feasibility of improving positioning accuracy by utilizing the temporal correlation of RSS was proven theoretically. In research by Singh [12], for distributed and isotropic WSNs, only a single anchor node was regarded as a reference node and the concept of virtual anchor node projection was proposed, which solved the problem of line-of-sight occlusion in the localization process.

Accurate distance measurement between node pairs is the basis for node positioning by maximum likelihood (ML), least squares (LS), MDS, and other positioning algorithms. However, in the actual ranging process, due to factors such as energy constraints or noise, the distance measurements between node pairs are missing or imprecise. Consequently, the positioning accuracy of the above algorithms is reduced.

In response to the above problems, the butterfly optimization algorithm was introduced in Arora’s research [13] to solve the problem of WSN localization under Gaussian noise interference. Fang [7] focused on the use of adaptive Kalman filtering to eliminate the influence of measurement noise and a localization algorithm based on MDS and adaptive Kalman filtering was proposed, which realized node localization with high positioning accuracy and low time complexity. More recent examinations by Fang [10], due to the weighted k-nearest neighbor algorithm, cannot be applied to estimate the node position in a noise environment. Based on adaptive Kalman filtering and the meme algorithm, an optimal weighted k-nearest neighbor algorithm for WSN fingerprint localization was proposed. Following this, and considering the influence of the multipath effect, the path loss and fading models of various multimedia and multipath communication scenarios in the network were given in Sahota’s work [14] and the received signal strength was modeled according to the transmission distance and the position coordinates of the nodes. Based on the maximum likelihood optimization, the derived statistical model was used to achieve node positioning.

However, while all of the above works strove to reduce the influence of noise on positioning accuracy, the detection and separation of noise in the distance measurement were not involved. In Feng’s research [15], the theory of matrix completion was introduced into the localization of a wireless sensor and the localization problem was transformed into an issue of low-rank matrix recovery. However, in this paper, the Gaussian noise was taken merely as the measurement noise and the composite noise was not taken into account, which leads to low positioning accuracy. Additionally, in Guo’s work [11], considering the influence of Gaussian noise and outlier noise on the EDM, a weighted semi-definite relaxation localization method was derived based on SDP, which in turn was based on a low-rank matrix completion algorithm by using the semi-definite embedding theorem to improve the accuracy of node localization. However, due to the high complexity of the algorithm, it is not suitable for dealing with large-scale WSN localization. In research by Xiao [16], Gaussian noise and outlier noise were considered simultaneously as composite noise and the localization accuracy was improved. Regrettably, the neglect of the pulse noise gave rise to unsatisfactory positioning accuracy. Given the above situation, we designed and implemented a robust and efficient WSN localization algorithm based on regularization matrix completion and the extended linear Bregman iterative method, to eliminate the impact of Gaussian noise, outlier noise, and pulse noise on positioning accuracy.

This paper mainly examines range-based localization technology, which utilizes the a priori physical position coordinates of beacon nodes and the distance measurement between node pairs to locate the unknown nodes in a WSN. In reality, two challenges hinder the application of this technology: (1) Due to factors such as environmental and energy constraints, the distance measurements between quite a few node pairs are missing; and (2) in the actual ranging process, the ranging accuracy will be affected by composite noise that is composed of Gaussian noise, outlier noise, and pulse noise. The limitations of the hardware give rise to Gaussian noise that obeys Gaussian distribution. Outlier noise results in the multipath effect or a malicious attack and follows Laplacian distribution. Additionally, uncertainty in the environment and the malfunction of a few sensor nodes lead to continuous errors which obey the Laplacian distribution, namely pulse error appearing in the form of a partial continuous mistake in the row or column of the EDM. The number of consecutive errors is called the width of the pulse noise.

Through the above analysis, it can be perceived that the observation matrix (a distance matrix constructed from distance measurements between node pairs in the real world) has data missing, as well as being contaminated by composite noise. Accordingly, it cannot be directly used in node localization. The matrix completion technique is in line with this demand. Therefore, an army of algorithms have been proposed one after another based on matrix completion and some have taken into account the influence of Gaussian noise and outlier noise [11,16,17]. However, due to the neglect of pulse noise by existing algorithms, positioning accuracy still needs to be further improved.

To overcome the above problems of missing and corrupted data, a WSN localization algorithm was proposed based on distance measurement. Due to the natural low-rank character of the EDM, the problem of EDM recovery is transformed into an issue of matrix completion under the condition of composite noise. Meanwhile, pulse noise is smoothed by $L_{1,2}$-norm based on the regularization technique. Aiming at solving the problem effectively, we extended the linear Bregman iterative algorithm in vector space to multidimensional space, and based on Bregman divergence, we designed a robust and efficient localization algorithm via matrix completion (LBDMC) by the multidimensional scaling (MDS) method.

The primary contributions of this paper are as follows:

1. We establish a novel matrix completion model employing the regularization technique for EDM recovery in WSNs. The model achieves a superior performance under pulse noise, as well as Gaussian noise and outlier noise.
2. In order to maintain the low-rank character and sparsity of the matrix variables while improving the stability of the model, we propose a robust and efficient algorithm named LBDMC by introducing the linear Bregman iterative method. The experimental results show that LBDMC has high positioning accuracy and excellent scalability, which are superior to the existing localization algorithms.
3. LBDMC can accurately acquire the location information contaminated by outliers and pulse noise in the observation matrix and then can determine the fault nodes, which can provide a basis for the fault diagnosis of the nodes in WSNs to a certain extent.

The remainder of this paper is organized as follows. Section 2 introduces the matrix completion technique and Bregman divergence. Section 3 outlines the problem formulation. The matrix completion algorithm based on Bregman divergence to complete EDM recovery is presented in Section 4 and based on this, the WSN localization is realized by MDS technology. Section 5 introduces the numerical experiments and analyzes the experimental results. Finally, the content of this paper is summarized.

## 2. Related Work

### 2.1. Matrix Completion Technique

The matrix completion (MC) technique is a generalization of compressed sensing in matrix space, which is devoted to solving the problem of recovery of missing elements in two-dimensional space. In general, the matrix completion problem can be described as the following minimized constraint model [18]:

(1) $$\underset{X}{\min}\;rank\left(X\right)\;s.t.P_{\Omega }\left(M\right)=P_{\Omega }\left(X\right)$$

where, $X,M\in {\mathbb{R}}^{m\times n}$ denote the target matrix to be recovered and the observation matrix, respectively. rank(⋅) denotes the rank function of the matrix. $P_{\Omega }\left(\cdot \right)$ represents an orthogonal projection operator, which is defined as:

(2) $${\left[P_{\Omega }\left(M\right)\right]}_{ij}=\left\{ \begin{array}{l} M_{ij}\;\left(i,j\right)\in \Omega \\ 0\;\;otherwise \end{array}\right.$$

where Ω∈[1:m]×[1:n] denotes the index set of elements.

However, since the rank function is nonconvex and nonsmooth, Equation (1) is loosened to the following constrained convex optimization model:

(3) $$\underset{X}{\min}\;{\left\|X\right\|}_{*}\;s.t.P_{\Omega }\left(M\right)=P_{\Omega }\left(X\right)$$

where ${\left\|X\right\|}_{*}=\sum \sigma \left(X\right)$ denotes the nuclear norm of matrix X, and σ(X) are singular values of the matrix X. However, in practice, considering that the observation matrix is usually corrupted by noise, Equation (3) is further modified as [19]:

(4) $$\underset{X}{\min}\;{\left\|X\right\|}_{*}\;s.t.X+E=M,P_{\Omega }\left(E\right)=0$$

where $E\in {\mathbb{R}}^{m\times n}$ denotes the noise matrix.

Regarding Equation (4), various optimization algorithms have been proposed. These mainly include SVT (Singular Value Thresholding) [19], IALM (Inexact Augmented Lagrange Multiplier) [20], FPCA (Fixed Point Continuation with Approximate SVD) [21], OptSpace [22], ScGrassMC [23], and so forth. IALM regards MC as a special case of the Robust Principal Component Analysis problem, and the quadratic penalty term is utilized to enhance the traditional Lagrange function, which allows each variable to be updated in closed form. OptSpace is essentially a gradient descent algorithm, which is constrained by low-rank character so that the matrix elements obtained by MC are as close as possible to the actual values. The problem is that the rank information of the matrix needs to be estimated when the rank is unknown. ScGrassMC introduced a non-canonical metric on the Grassmann manifold to improve OptSpace. Unfortunately, all of the above algorithms are only able to recover the target matrix from the observation matrix, which is damaged only by Gaussian noise and outlier noise. Therefore, when the observation matrix is disturbed by pulse noise, the recovery accuracy is not satisfactory—these algorithms are sensitive to pulse noise.

### 2.2. Bregman Divergence

As an optimization algorithm, the linear Bregman iteration is widely used in the fields of compressed sensing [24], image de-noising [25], target detection [26], and quantitative clustering [27]. It has been one of the most effective methods for solving norm optimization problems.

**Definition** **1.** ***Bregman Divergence** [28]. Let* $J\left(x\right):{\mathbb{R}}^{n}\to \mathbb{R}$ *be a continuously-differentiable convex function,* ∀u,v∈x *. The Bregman divergence of the function* J *between two points* u *and* v *is defined as:*

(5) $$D_{J}^{p}\left(u,v\right)=J\left(u\right)-J\left(v\right)-\langle p,u-v\rangle \;$$

*where* p∈∂J(v) *denotes a sub-gradient of the function* J *at the point* v *, and* ∂J(v) *is the sub-differential of function* J *at point* v *, which is the set of sub-gradients* p *.*

**Definition** **2.** ***Multivariate Functions Bregman Divergence.** Let*$J\left(X_{\left(1\right)},X_{\left(2\right)},\cdots ,X_{\left(l\right)}\right):{\mathbb{R}}^{n}\to \mathbb{R}$*be a continuously-differentiable convex function,*$\forall x_{\left(i\right)},v_{\left(i\right)}\in X_{\left(i\right)},i=1,2,\cdots l$*. The multivariate function Bregman divergence of*J*between two points*$\left(u_{\left(1\right)},u_{\left(2\right)},\cdots ,u_{\left(l\right)}\right)$*and*$\left(v_{\left(1\right)},v_{\left(2\right)},\cdots ,v_{\left(l\right)}\right)$*is defined as:*

(6) $$\begin{array}{l} D_{J}^{p}\left[\left(u_{\left(1\right)},u_{\left(2\right)},\cdots ,u_{\left(l\right)}\right),\left(v_{\left(1\right)},v_{\left(2\right)},\cdots ,v_{\left(l\right)}\right)\right]\\ =J\left(u_{\left(1\right)},u_{\left(2\right)},\cdots ,u_{\left(l\right)}\right)-J\left(v_{\left(1\right)},v_{\left(2\right)},\cdots ,v_{\left(l\right)}\right)-\sum_{i=1}^{l}\langle p_{v_{\left(i\right)}},u_{\left(i\right)}-v_{\left(i\right)}\rangle \end{array}$$

*where*$p=\left(p_{v_{\left(1\right)}},p_{v_{\left(2\right)}},\cdots p_{v_{\left(l\right)}}\right)\in \partial J\left(v_{\left(1\right)},v_{\left(2\right)},\cdots ,v_{\left(l\right)}\right)$*denotes a sub-gradient of the multivariate function*J*at the point*$\left(v_{\left(1\right)},v_{\left(2\right)},\cdots ,v_{\left(l\right)}\right)$.

Here are three examples of multivariate functions J. The corresponding multivariate function Bregman divergence is given. ($D_{J}^{p}\left[\left(u_{\left(1\right)},u_{\left(2\right)},\cdots ,u_{\left(l\right)}\right),\left(v_{\left(1\right)},v_{\left(2\right)},\cdots ,v_{\left(l\right)}\right)\right]$ is shorthand for $D_{J}^{p}\left(U,V\right)$).

1. $J\left(x_{\left(1\right)},x_{\left(2\right)},\cdots ,x_{\left(l\right)}\right)=\sum_{i=1}^{l}{\left\|x_{\left(i\right)}\right\|}^{2}$, in which, the Euclidean model $\left\|x_{\left(i\right)}\right\|:=\sqrt{\langle x_{\left(i\right)},x_{\left(i\right)}\rangle }$.
  (7) $$D_{J}^{p}\left(U,V\right)=\sum_{i=1}^{l}{\left\|u_{\left(i\right)}\right\|}^{2}-\sum_{i=1}^{l}{\left\|v_{\left(i\right)}\right\|}^{2}-\sum_{i=1}^{l}\langle 2v_{\left(i\right)},u_{\left(i\right)}-v_{\left(i\right)}\rangle =\sum_{i=1}^{l}{\left\|u_{\left(i\right)}-v_{\left(i\right)}\right\|}^{2}$$
  when l=1, $D_{J}^{p}\left(U,V\right)$ is the square of our most commonly used Euclidean distance.
2. $J\left(x_{\left(1\right)},x_{\left(2\right)},\cdots ,x_{\left(l\right)}\right)=\sum_{i=1}^{l}x_{\left(i\right)}{}^{T}Ax_{\left(i\right)}$.
  (8) $$D_{J}^{p}\left(U,V\right)=\sum_{i=1}^{l}{\left(u_{\left(i\right)}-v_{\left(i\right)}\right)}^{T}A\left(u_{\left(i\right)}-v_{\left(i\right)}\right)\;$$
  when *l* = 1, $D_{J}^{p}\left(U,V\right)$ is the Mahalanobis distance.
3. $J\left(x_{\left(1\right)},x_{\left(2\right)},\cdots ,x_{\left(l\right)}\right)=\sum_{i=1}^{l}\sum_{j=1}^{n}\left(x_{\left(i\right)j}\ln x_{\left(i\right)j}\right),x_{\left(i\right)}\in {\mathbb{R}}^{n}$.
  (9) $$D_{J}^{p}\left(U,V\right)=\sum_{i=1}^{l}\sum_{j=1}^{n}\left(u_{\left(i\right)j}\ln\frac{u_{\left(i\right)j}}{v_{\left(i\right)j}}\right)$$
  when *l* = 1, $D_{J}^{p}\left(U,V\right)$ is the KL divergence.

## 3. Problem Formulation

**Figure 1:**
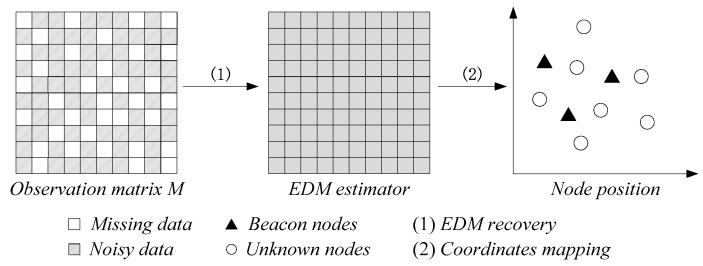
Localization for a wireless sensor network (WSN). The number of nodes is set to 10. In addition, there are three beacon nodes in the WSN.

For a WSN disposed in a certain *d*-dimensional area S ($S\in {\mathbb{R}}^{d}$), we suppose that n nodes are deployed randomly in S, if $X=\left[x_{1},x_{2},\cdots .x_{n}\right]$ ($X\in {\mathbb{R}}^{d\times n}$ denotes the coordinates matrix of *n* nodes in *d*-dimensional space), then the Euclidean distance matrix $R\in {\mathbb{R}}^{n\times n}$ can be obtained ($R_{ij}={\left\|x_{i}-x_{j}\right\|}^{2},i,j=1,2,\cdots ,n$). The observation matrix of EDM $M\in {\mathbb{R}}^{n\times n}$ between nodes is measured from R. As mentioned above, the matrix M is incomplete and noisy. After that, we divide WSN localization into two stages: (1) EDM recovery and (2) coordinates mapping, as shown in Figure 1. Due to the incompleteness and noise-containing property of the matrix M, the MDS-based localization method cannot realize the WSN localization with high accuracy. It is indispensable to complete the accurate estimation of R based on the matrix completion technique.

•  Stage 1: EDM Recovery.

The proof of rank(R)≤d+2 has been given in Fu’s work [29]; therefore, in the case of n>>d, the R is a low-rank matrix. However, the observation matrix is usually contaminated by pulse noise. Consequently, the problem of EDM recovery can then be formulated as the following matrix completion model:

(10) $$\begin{array}{l} \underset{R,G,O,C\in {\mathbb{R}}^{n\times n}}{\min}\;\;{\left\|R\right\|}_{*}+\varphi {\left\|G\right\|}_{F}^{2}+\mu {\left\|O\right\|}_{1}+\lambda {\left\|C\right\|}_{1,2}\\ \;\;s.t.\;P_{\Omega }\left(M\right)=P_{\Omega }\left(R+G+O+C\right) \end{array}$$

where G,O,C denotes the Gaussian noise matrix, outlier noise matrix, and pulse noise matrix, respectively. φ,μ,λ is a tunable parameter for balancing three kinds of noise. ${\left\|G\right\|}_{F}=\sqrt{\sum_{i=1}^{n}\sum_{j=1}^{n}{\left|G_{ij}\right|}^{2}}$ denotes the Frobenius norm of the Gaussian noise matrix. ${\left\|O\right\|}_{1}=\sum_{i=1}^{n}\sum_{j=1}^{n}\left|O_{ij}\right|$ denotes the $L_{1}$-norm of the outlier noise matrix. ${\left\|C\right\|}_{1,2}=\sum_{i=1}^{n}\sqrt{\sum_{j=1}^{n}C_{ij}{}^{2}}$ denotes the $L_{1,2}$-norm of the pulse noise matrix.

The EDM estimator $\hat{R}$($\hat{R}\in {\mathbb{R}}^{n\times n}$) can be obtained by solving Equation (10).

•  Stage 2: Coordinates Mapping

On the basis of obtaining the EDM estimator $\hat{R}$ in Stage 1, we can employ the MDS-based localization algorithm to obtain the node coordinates. The specific steps are: first, according to $\hat{R}$, the relative coordinates of the sensor nodes can be generated. Moreover, the coordinates mapping matrix can be calculated by aligning the coordinates of three or more beacon nodes. Lastly, per the coordinates mapping matrix, the relative coordinates can be mapped to the absolute coordinates.

If we suppose that there are k (k≥3) beacon nodes, and $L_{t},T_{t}\in {\mathbb{R}}^{d\times 1}\left(t=1,2,\cdots ,k\right)$ denotes the relative coordinates and absolute coordinates of the tth beacon node, respectively, then the coordinates mapping matrix Q is:

(11) $$Q=\frac{\left[T_{2}-T_{1},T_{3}-T_{1},\cdots ,T_{k}-T_{1}\right]}{\left[L_{2}-L_{1},L_{3}-L_{1},\cdots ,L_{k}-L_{1}\right]}$$

The absolute coordinates of the nodes in the entire WSN can be calculated as:

(12) $$\left\{T|T_{i}-T_{1}=Q\times \left(L_{i}-L_{1}\right),i=1,2,\cdots ,n\right\}$$

## 4. Localization Algorithm via Matrix Completion Based on Bregman Divergence

### 4.1. BDMC Algorithm

In this section, we introduce Bregman divergence to solve the matrix completion model. The augmented Lagrangian function corresponding to Equation (10) is:

(13) $$\begin{array}{l} L_{\rho }\left(R,G,O,C,Y\right)={\left\|R\right\|}_{*}+\varphi {\left\|G\right\|}_{F}^{2}+\mu {\left\|O\right\|}_{1}+\lambda {\left\|C\right\|}_{1,2}\\ +\langle Y,R+G+O+C-M\rangle +\frac{\rho }{2}{\left\|M-\left(R+G+O+C\right)\right\|}_{F}^{2} \end{array}$$

where $Y\in {\mathbb{R}}^{n\times n}$ denotes the Lagrangian multiplier. ρ>0 denotes the tunable parameter whose size is negatively correlated with the Gaussian noise term. If ρ is set to a large value, the purpose of implicitly smoothing the Gaussian noise can be achieved. Thus, Equation (10) can be simplified to the following:

(14) $$\begin{array}{l} \underset{R,O,C\in {\mathbb{R}}^{n\times n}}{\min}\;{\left\|R\right\|}_{*}+\mu {\left\|O\right\|}_{1}+\lambda {\left\|C\right\|}_{1,2}\\ \;\;s.t.\;\;P_{\Omega }\left(M\right)=P_{\Omega }\left(R+O+C\right) \end{array}$$

In order to solve Equation (14) effectively, we relax it into the following unconstrained optimization problems:

(15) $$\underset{R,O,C\in {\mathbb{R}}^{n\times n}}{\min}\;\tau \left({\left\|R\right\|}_{*}+\mu {\left\|O\right\|}_{1}+\lambda {\left\|C\right\|}_{1,2}\right)+\frac{1}{2}{\left\|P_{\Omega }\left(M-\left(R+O+C\right)\right)\right\|}_{F}^{2}$$

where τ>0.

Furthermore, an outstanding model should be stable and scalable. Stability means that the well-trained model should not change much with different training sets. Moreover, scalability means the model can be applied to various situations. The Bregman iteration is a method to enhance the stability and scalability. For the convenience of description, let:

(16) $$\begin{array}{l} J\left(R,O,C\right)=\tau \left({\left\|R\right\|}_{*}+\mu {\left\|O\right\|}_{1}+\lambda {\left\|C\right\|}_{1,2}\right)\\ H\left(R,O,C\right)=\frac{1}{2}{\left\|P_{\Omega }\left(M-\left(R+O+C\right)\right)\right\|}_{F}^{2} \end{array}$$

Then the Equation (15) is equivalent to:

(17) $$\underset{R,O,C\in {\mathbb{R}}^{n\times n}}{\min}\;J\left(R,O,C\right)+H\left(R,O,C\right)$$

The multivariate function Bregman divergence is introduced to solve Equation (17). According to Definition 2, the Bregman divergence of the function J between the two points (R,O,C) and $\left(R^{k},O^{k},C^{k}\right)$ is:

(18) $$D_{J}^{P^{k}}\left[\left(R,O,C\right),\left(R^{k},O^{k},C^{k}\right)\right]=J\left(R,O,C\right)-J\left(R^{k},O^{k},C^{k}\right)-A$$

where $A=\langle \tau P_{R}^{k},R-R^{k}\rangle +\langle \tau \mu P_{O}^{k},O-O^{k}\rangle +\langle \tau \lambda P_{C}^{k},C-C^{k}\rangle$, $P^{k}=\left(P_{R}^{k},P_{O}^{k},P_{C}^{k}\right)$ $\in \partial J\left(R^{k},O^{k},C^{k}\right)$ denotes a sub-gradient of the function J at the point $\left(R^{k},O^{k},C^{k}\right)$.

Therefore, Equation (17) can be iteratively solved as follows:

(19) $$\left\{ \begin{array}{l} \left(R^{k+1},O^{k+1},C^{k+1}\right)=\underset{R,O,C\in {\mathbb{R}}^{n\times n}}{\arg\min}\;D_{J}^{P^{k}}\left(\left(R,O,C\right),\left(R^{k},O^{k},C^{k}\right)\right)+H\left(R,O,C\right)\\ 0\in \partial {\left(D_{J}^{P^{k}}\left(\left(R,O,C\right),\left(R^{k},O^{k},C^{k}\right)\right)+H\left(R,O,C\right)\right)|}_{R^{k+1}}\\ 0\in \partial {\left(D_{J}^{P^{k}}\left(\left(R,O,C\right),\left(R^{k},O^{k},C^{k}\right)\right)+H\left(R,O,C\right)\right)|}_{O^{k+1}}\\ 0\in \partial {\left(D_{J}^{P^{k}}\left(\left(R,O,C\right),\left(R^{k},O^{k},C^{k}\right)\right)+H\left(R,O,C\right)\right)|}_{C^{k+1}} \end{array}\right.$$

Inspired by the idea of a split Bregman iteration [30], extending it to the matrix space as well as applying the alternating minimization method, Equation (17) can be solved further by SBI-AM (described in Algorithm 1).

**Algorithm 1** Algorithmic description of the SBI-AM**Input:**$P_{\Omega }\left(M\right)$, the maximum number of iterations N
**Output:**
$R^{opt},O^{opt},C^{opt}$
1: **Initialize**
$O^{0}=C^{0}=0$, $P_{O}^{0}=P_{C}^{0}=0$.2: **for**
*k = 0 to N*3:  $R^{k+1}=\underset{R\in {\mathbb{R}}^{n\times n}}{\arg\min}\;\tau {\left\|R\right\|}_{*}-\tau \langle P_{R}^{k},R\rangle +\frac{1}{2}{\left\|P_{\Omega }\left(M-R-O-C\right)\right\|}_{F}^{2}$4:  $O^{k+1}=\underset{O\in {\mathbb{R}}^{n\times n}}{\arg\min}\;\tau \mu {\left\|O\right\|}_{1}-\tau \mu \langle P_{O}^{k},O\rangle +\frac{1}{2}{\left\|P_{\Omega }\left(M-R^{k+1}-O-C\right)\right\|}_{F}^{2}$5:  $C^{k+1}=\underset{C\in {\mathbb{R}}^{n\times n}}{\arg\min}\;\tau \lambda {\left\|C\right\|}_{1,2}-\tau \lambda \langle P_{C}^{k},C\rangle +\frac{1}{2}{\left\|P_{\Omega }\left(M-R^{k+1}-O^{k+1}-C\right)\right\|}_{F}^{2}$6:  $P_{R}^{k+1}=P_{R}^{k}+\frac{1}{\tau }P_{\Omega }\left(M-R^{k+1}-O^{k+1}-C^{k+1}\right)$7:  $P_{O}^{k+1}=P_{O}^{k}+\frac{1}{\tau \mu }P_{\Omega }\left(M-R^{k+1}-O^{k+1}-C^{k+1}\right)$8:  $P_{C}^{k+1}=P_{C}^{k}+\frac{1}{\tau \lambda }P_{\Omega }\left(M-R^{k+1}-O^{k+1}-C^{k+1}\right)$9: **end for**10: **return**
$R^{opt}\leftarrow R^{N+1},O^{opt}\leftarrow O^{N+1},C^{opt}\leftarrow C^{N+1}$

It is not difficult to see from the SBI-AM algorithm that, since the functions ${\left\|R\right\|}_{*}$, ${\left\|O\right\|}_{1}$ and ${\left\|C\right\|}_{1,2}$ are not differentiable, steps 2–4 in the algorithm cannot directly solve the corresponding variables. Accordingly, we have the following basic definitions and theorems.

**Definition** **3** [31]. ***Proximal Operator.** Let* g(X) *be a real-value convex function defined on* ${\mathbb{R}}^{m\times n}$*,* τ>0*,* $\forall Z\in {\mathbb{R}}^{m\times n}$*, then the proximal operator of* g(X) is defined as:

(20) $$prox_{\tau g\left(X\right)}\left(Z\right)=\underset{X\in {\mathbb{R}}^{m\times n}}{\arg\min}\left(\tau g\left(X\right)+\frac{1}{2}{\left\|X-Z\right\|}_{F}^{2}\right)$$

**Theorem** **1** [32]. *Let* $F_{1},F_{2}$ *be lower semicontinuous and convex functions defined on* ${\mathbb{R}}^{m\times n}$ *such that* $F_{2}$ *is differentiable on a* ${\mathbb{R}}^{m\times n}$ *with a* β−Lipschitz *continuous gradient. For a convex optimization problem* $\underset{X\in {\mathbb{R}}^{m\times n}}{\min}\;F_{1}\left(X\right)+F_{2}\left(X\right)$*, if* $F_{1}+F_{2}$ *is coercive and strictly convex, the solution of the problem takes on uniqueness. For an arbitrary initial value* $X_{0}$*,* ∀0<δ<2/β*, the iterative sequence* $X_{k+1}$ *generated by the following statement can converge to the unique solution of the problem.*

(21) $$X_{k+1}=\underset{X\in {\mathbb{R}}^{m\times n}}{\arg\min}\left(\delta F_{1}\left(X\right)+\frac{1}{2}{\left\|X-\left(X_{k}-\delta \nabla F_{2}\left(X_{k}\right)\right)\right\|}_{F}^{2}\right)$$

**Theorem** **2** [19]. *For* ∀κ>0*,* $Z\in {\mathbb{R}}^{m\times n}$*, the proximal operator of the nuclear norm of matrix* X*,* $prox_{\kappa {\left\|X\right\|}_{*}}\left(Z\right)$*, is*

(22) $$prox_{\kappa {\left\|X\right\|}_{*}}\left(Z\right)=D_{\kappa }\left(Z\right)$$

*where* $D_{\kappa }\left(\cdot \right)$ *denotes the soft-thresholding operator [28].*

**Theorem** **3** [33]. *For* ∀κ>0*,* $Z\in {\mathbb{R}}^{m\times n}$*, the proximal operator of the* $L_{1}$*-norm of matrix* X*,* $prox_{\kappa {\left\|X\right\|}_{1}}\left(Z\right)$*, is:*

(23) $$prox_{\kappa {\left\|X\right\|}_{1}}\left(Z\right)=S_{\kappa }\left(Z\right)$$

*where* $S_{\kappa }\left(\cdot \right)$ *denotes the shrinkage operator [28].*

**Theorem** **4** [34]. *For* ∀κ>0*,* $Z\in {\mathbb{R}}^{m\times n}$*, the proximal operator of the* $L_{1,2}$*-norm of matrix* X*,* $prox_{\kappa {\left\|X\right\|}_{1,2}}\left(Z\right)$*, is:*

(24) $$prox_{\kappa {\left\|X\right\|}_{1,2}}\left(Z\right)=J_{\kappa }\left(Z\right)$$

*where* $J_{\kappa }{\left(Z\right)}_{\left(i\right)}=\max\left\{1-\kappa /{\left\|Z_{\left(i\right)}\right\|}_{2},0\right\}\cdot Z_{\left(i\right)},i=1,2,\cdots ,m$.

Applying the above definitions and theorems, the initialization of variables is set to $R^{0}=0$, $O^{0}=0$, $C^{0}=0$, $P_{O}^{0}=0$, $P_{C}^{0}=0$, and the update steps of $\left(R^{k+1},O^{k+1},C^{k+1}\right)$ are listed as follows:

- Step 1. Update *R*
  According to Definition 3 and Theorem 1, $R^{k+1}$ can be rewritten as:
  (25) $$R^{k+1}=prox_{\tau \delta {\left\|R\right\|}_{*}-\tau \delta \langle P_{R}^{k},R\rangle }\left(R^{k}+\delta P_{\Omega }\left(M-R^{k}-O^{k}-C^{k}\right)\right)$$
  Let $\Gamma_{R}^{k}=\delta P_{\Omega }\left(M-R^{k}-O^{k}-C^{k}\right)$, and Equation (25) is simplified to:
  (26) $$R^{k+1}=\underset{R}{\arg\min}\;\tau \delta {\left\|R\right\|}_{*}+\frac{1}{2}{\left\|R-R^{k}-\tau \delta P_{R}^{k}-\Gamma_{R}^{k}\right\|}_{F}^{2}\;$$
  Meanwhile, we can deduce the iterative formula of $P_{R}$:
  (27) $$P_{R}^{k+1}=P_{R}^{k}-\frac{1}{\tau \delta }\left(R^{k+1}-R^{k}-\Gamma_{R}^{k}\right)$$
  Furthermore, let:
  (28) $$B^{k}=R^{k}+\tau \delta P_{R}^{k}+\Gamma_{R}^{k}$$
  Obviously, the iterative formula of B is:
  (29) $$B^{k}-B^{k-1}=\delta P_{\Omega }\left(M-R^{k}-O^{k}-C^{k}\right)$$
  Then, Equation (26) can be reformulated as:
  (30) $$R^{k+1}=\underset{R}{\arg\min}\;\tau \delta {\left\|R\right\|}_{*}+\frac{1}{2}{\left\|R-B^{k}\right\|}_{F}^{2}$$
  According to Theorem 2:
  (31) $$R^{k+1}=D_{\tau \delta }\left(B^{k}\right)$$
- Step 2. Update *O*
  Similar to Step 1, for outlier noise matrix O:
  (32) $$O^{k+1}=\underset{O}{\arg\min}\;\tau \mu \delta {\left\|O\right\|}_{1}+\frac{1}{2}{\left\|O-O^{k}-\tau \mu \delta P_{O}^{k}-\Gamma_{O}^{k}\right\|}_{F}^{2}\;$$
  where $\Gamma_{O}^{k}=\delta P_{\Omega }\left(M-R^{k+1}-O^{k}-C^{k}\right)$.
  Let $I^{k}=O^{k}+\tau \mu \delta P_{O}^{k}+\Gamma_{O}^{k}$, and we can update O as:
  (33) $$O^{k+1}=\underset{O}{\arg\min}\;\tau \mu \delta {\left\|O\right\|}_{1}+\frac{1}{2}{\left\|O-I^{k}\right\|}_{F}^{2}\;$$
  (34) $$I^{k}-I^{k-1}=\delta P_{\Omega }\left(M-R^{k+1}-O^{k}-C^{k}\right)\;$$
  Based on Theorem 3, the analytical solution of (34) is:
  (35) $$O^{k+1}=S_{\tau \mu \delta }\left(I^{k}\right)\;$$
- Step 3. Update *C*
  Similarly, for pulse noise matrices C:
  (36) $$C^{k+1}=\underset{C}{\arg\min}\;\tau \lambda \delta {\left\|C\right\|}_{1,2}+\frac{1}{2}{\left\|C-C^{k}-\tau \lambda \delta P_{C}^{k}-\Gamma_{C}^{k}\right\|}_{F}^{2}\;$$
  where $\Gamma_{C}^{k}=\delta P_{\Omega }\left(M-R^{k+1}-O^{k+1}-C^{k}\right)$.
  Let $U^{k}=C^{k}+\tau \lambda \delta P_{C}^{k}+\Gamma_{C}^{k}$, and we can update C as:
  (37) $$C^{k+1}=\underset{C}{\arg\min}\;\tau \lambda \delta {\left\|C\right\|}_{1,2}+\frac{1}{2}{\left\|C-U^{k}\right\|}_{F}^{2}\;$$
  (38) $$U^{k}=U^{k-1}+\delta P_{\Omega }\left(M-R^{k+1}-O^{k+1}-C^{k}\right)\;$$
  According to Theorem 4, Equation (39) can be solved as:
  (39) $$C^{k+1}=J_{\tau \lambda \delta }\left(U^{k}\right)\;$$

In Algorithm 2 we sum up the above steps and the whole optimization process of solving Equation (10) can be summarized as the BDMC algorithm demonstrated in Algorithm 2.

**Algorithm 2** Algorithmic description of BDMC**Input**: $P_{\Omega }\left(M\right)$, τ,μ,λ,δ, the maximum number of iterations *N***Output**: $R^{opt},O^{opt},C^{opt}$1: **Initialize**
$R^{0}=0$, $O^{0}=0,C^{0}=0$, $B^{-1}=0,I^{-1}=0,U^{-1}=0$.2: **for**
*k=0 to N*3: $B^{k}=B^{k-1}+\delta P_{\Omega }\left(M-R^{k}-O^{k}-C^{k}\right)$.4: $R^{k+1}=D_{\tau \delta }\left(B^{k}\right)$.5: $I^{k}=I^{k-1}+\delta P_{\Omega }\left(M-R^{k+1}-O^{k}-C^{k}\right)$.6: $O^{k+1}=S_{\tau \mu \delta }\left(I^{k}\right)$.7: $U^{k}=U^{k-1}+\delta P_{\Omega }\left(M-R^{k+1}-O^{k+1}-C^{k}\right)$.8: $C^{k+1}=J_{\tau \lambda \delta }\left(U^{k}\right)$.9: **end for**10: **return**
$R^{opt}\leftarrow R^{N+1},O^{opt}\leftarrow O^{N+1},C^{opt}\leftarrow C^{N+1}$

In the iterative process of Algorithm 2, it can be observed that, on the one hand, BDMC can maintain R with low rank. On the other hand, it can maintain the sparsity of (B,I,U) to save storage space. Each iteration only involves the partial singular value decomposition (SVD) of a sparse matrix, while a mature PROPACK software package can be used for the partial SVD of large sparse matrices. These features ensure the scalability and efficiency of BDMC.

### 4.2. LBDMC Algorithm

The EDM estimator $\hat{R}$ obtained by the BDMC algorithm achieves the goal of EDM recovery, which means the relative coordinate is available. Consequently, combined with BDMC, the MDS-based localization method is used for WSN localization. We summarize and give the detailed steps of WSN Localization via matrix completion based on Bregman divergence (LBDMC) in Algorithm 3.

**Algorithm 3** Algorithmic description of LBDMC**Input:**$P_{\Omega }\left(M\right)$, τ,μ,λ,δ, the maximum number of iterations N,the coordinates of the beacon nodes $\left\{T_{1},T_{2},\cdots ,T_{k}|k\geq 3\right\}$.**Output:** the absolute coordinates of nodes in the entire WSN $\left\{T_{i}|i=1,2,\cdots ,n\right\}$./* EDM recovery*/1: Compute EDM estimator $\hat{R}$ from data missing and noisy matrix M based on BDMC./*Node positioning based on MDS method*/2: $\left[U,\Lambda ,V\right]=svd\left(-\frac{1}{2}\Theta \hat{R}\Theta^{T}\right)$where $\Theta =Ι-\frac{1}{n}1\cdot 1^{T}$, Ι denotes the identity matrix.3: Generate relative coordinates.
$W=\Lambda_{d}^{1/2}U{\left(:,1:d\right)}^{T}$
where $W=\left[W_{1},W_{2},\cdots ,W_{n}\right]\in {\mathbb{R}}^{d\times n}$.4: Calculate the coordinates mapping matrix.
$Q=\frac{\left[T_{2}-T_{1},T_{3}-T_{1},\cdots T_{k}-T_{1}\right]}{\left[W_{2}-W_{1},W_{3}-W_{1},\cdots W_{k}-W_{1}\right]}$
5: Node coordinates mapping.
$\left\{T|T_{i}-T_{1}=Q\times \left(W_{i}-W_{1}\right),i=k+1,k+2,\cdots ,n\right\}$
6: **return**
T

## 5. Numerical Experiments and Results Analysis

In order to evaluate the efficacy of our proposed LBDMC, the EDM recovery errors, mean localization errors, localization errors variance, and localization errors’ cumulative distribution were selected as evaluation indicators and compared with IALM [20], OptSpace [22], and ScGrassMC [23]. We supposed that a WSN randomly distributed in a 100 m × 100 m square region with 100 nodes, few of which are beacon nodes and that the EDM is obtained according to distance measurements between the nodes. Then, we added noise to the EDM and randomly sampled the noisy EDM at the sampling rate to obtain the observation matrix as the training data of the above algorithm. At the same time, in order to avoid a contingency, we repeated the experiments 20 times and the average value was taken as the experimental results. Depending on the noise environment, we set the following four situations:

Case 1: The EDM is not contaminated by any noise; that is, the value in the observation matrix is accurate.

Case 2: The EDM is contaminated by Gaussian noise and outlier noise. We suppose that the Gaussian noise obeys a Gaussian distribution with a mean of 0 and a variance of 100. Meanwhile, the outlier noise obeys a Laplace distribution with a mean of 0 and a variance of 10,000.

Case 3: The EDM is corrupted by pulse noise. In the related experiments, we assume that the pulse noise, whose width is 30, obeys a Laplace distribution with a mean value of 0, and a variance of 10,000.

Case 4: The EDM is affected by Gaussian noise, outlier noise, and pulse noise. The Gaussian noise obeys a Gaussian distribution with a mean of 0 and a variance of 100. The outlier noise obeys a Laplace distribution with a mean of 0 and a variance of 10,000. In addition, the pulse noise, whose width is 30, obeys a Laplace distribution with mean value of 0 and a variance of 10,000.

### 5.1. Evaluation Indicators

We selected the following four indicators to evaluate the performance of the proposed LBDMC algorithm. Let $X\in {\mathbb{R}}^{2\times n}$, $R\in {\mathbb{R}}^{n\times n}$ (n=100 is the number of nodes) denote the node coordinates matrix and the EDM, respectively.

- EDM recovery errors REs:
  $$REs={\left\|\hat{R}-R\right\|}_{F}/{\left\|R\right\|}_{F}\;$$
  where $\hat{R}$ denotes the EDM estimator obtained by the BDMC algorithm.
- Mean localization errors LEs:
  $$LEs={\left\|\hat{X}-X\right\|}_{F}/n\;$$
  where $\hat{X}$ denotes the estimation of the node coordinate matrix X.
- Localization errors variance LEV:
  $$LEV=\frac{1}{n}\sum_{i=1}^{n}{\left(\Delta L_{i}-LEs\right)}^{2}\;$$
  where $\Delta L_{i}=\sqrt{{\left({\hat{x}}_{i}-x_{i}\right)}^{2}+{\left({\hat{y}}_{i}-y_{i}\right)}^{2}}$ denotes the localization errors of the i-th *node*, $\left(x_{i},y_{i}\right)$, $\left({\hat{x}}_{i},{\hat{y}}_{i}\right)$ i=1,2,⋯,n denote the coordinate of the i-th *node* and its estimator, respectively.
- Localization errors cumulative distribution LE_CDF:
  $$LE\_CDF=P\left(\Delta L_{i}\leq \sigma \right)\;$$
  where σ is a constant.

### 5.2. Comparison of Experiments

#### 5.2.1. Comparison of Convergence

**Figure 2:**
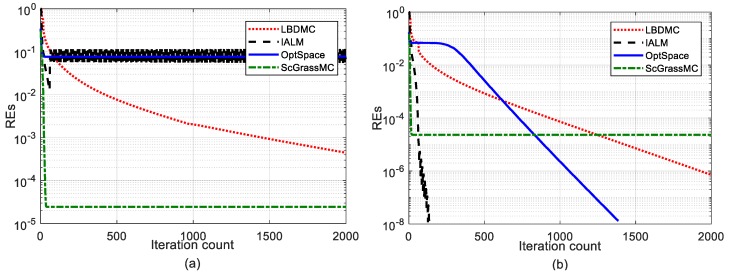
Comparison of convergence. The maximum number of iterations is fixed at 2000, and the tolerance is fixed at 10^−8^; that is, stopping iterations once *REs* < 10^−8^: (**a**) The sampling rate is fixed at 30%; (**b**) The sampling rate is fixed at 50%.

The convergence of the four algorithms at a sampling rate of 30% (a) and 50% (b) are plotted in Figure 2. It can be seen from Figure 2 that the convergence rate of the ScGrassMC algorithm was the fastest compared with the other three algorithms. In addition, compared to Figure 2a,b, the convergence rate of the ScGrassMC algorithm did not change much with the increase of the sampling rate, while the rest of the algorithms changes significantly. The IALM algorithm changed the most obviously; when the sampling rate was 50%, the convergence speed was second only to the ScGrassMC algorithm.

#### 5.2.2. Comparison of the EDM Recovery Errors

**Figure 3:**
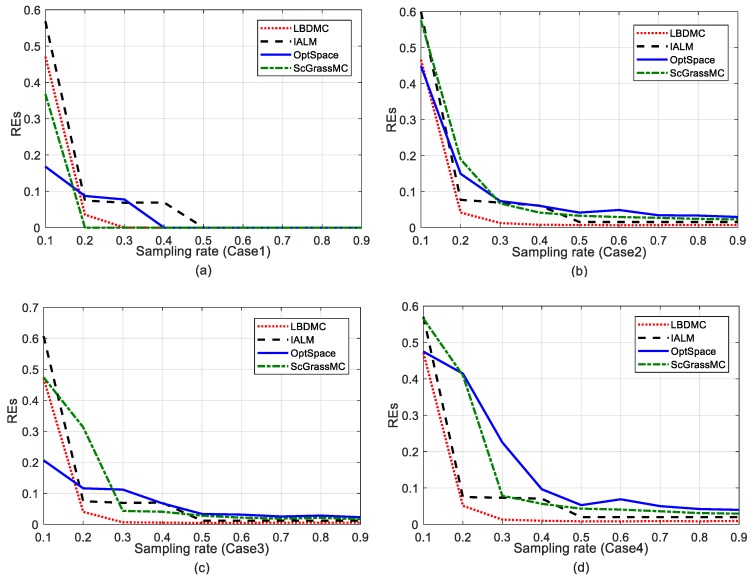
Comparison of EDM recovery errors under different noise conditions. The sampling rate varies successively from 10% to 90%: (**a**) EDM recovery errors under Case 1; (**b**) EDM recovery errors under Case 2; (**c**) EDM recovery errors under Case 3; (**d**) EDM recovery errors under Case 4.

EDM recovery errors under Case 1: The variations of the EDM recovery errors under different sampling rates are shown in Figure 3a. It can be observed that, under the noiseless condition, the recovery errors of each algorithm decreased rapidly with the increase of the sampling rate until they were close to zero. When the sampling rate was around 20%, the recovery errors of ScGrassMC could achieve an approximate zero and its performance was superior to the other three algorithms. However, the performance of the LBDMC and ScGrassMC was approximately the same when the sampling rate reached 30%, while IALM and OptSpace were relatively inferior.

EDM recovery errors under Case 2: The ratio of outlier noise (the outlier ratio) in each algorithm was set to 5%. As can be seen from Figure 3b, our proposed LBDMC outperformed the other three methods when the sampling rate was above 20% and could acquire an approximate zero recovery error with a 30% sampling rate. In contrast, the performances of OptSpace and ScGrassMC were obviously affected by noise, even if the sampling rate reached 90%. Therefore, the noise-tolerance of LBDMC under Case 2 was superior to the other three algorithms.

EDM recovery errors under Case 3: The ratio of pulse noise (the pulse ratio) in each algorithm is set to 10% (as an example, the row of the EDM corrupted by pulse noise). In Figure 3c, compared to Case 1, the performance of ScGrassMC under Case 3 obviously deteriorated, while the recovery errors of the LBDMC and IALM increased slightly. That is, LBDMC and IALM were pulse noise tolerant, and the others are not.

EDM recovery errors under Case 4: The outlier ratio in each algorithm was set to 5% and the pulse ratio was set to 10% (as an example, the row of the EDM corrupted by pulse noise). The EDM recovery errors under Case 4 are shown in Figure 3d. Since the EDM was contaminated by composite noise, the performance of OptSpace and ScGrassMC declined notably. In contrast, the LBDMC still acquired an approximate zero recovery error with a sampling rate above 30%.

#### 5.2.3. Comparison of Mean Localization Error and Error Variance

**Figure 4:**
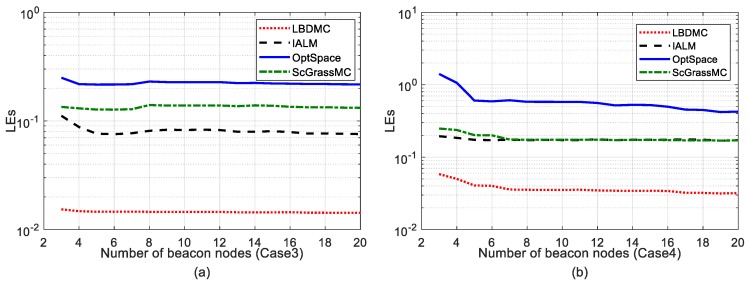
Mean localization error versus different number of beacons. The number of beacons varies successively from 3 to 20. The sampling rate is fixed at 50%: (**a**) Mean localization error under Case 3; (**b**) Mean localization error under Case 4.

Firstly, we investigated the effect of the number of beacon nodes on the mean localization errors and the sampling rate was fixed at 50%. Figure 4 shows the mean localization errors under Case 3a and Case 4b, respectively. In Figure 4, the mean localization error of each algorithm decreases as the number of beacon nodes increase. When the number of beacon nodes was less than six, the mean localization error varied more obviously. Consequently, we set the number of beacon nodes at six. In addition, we can observe that the mean localization error of the LBDMC was lower than that of the other three algorithms.

**Figure 5:**
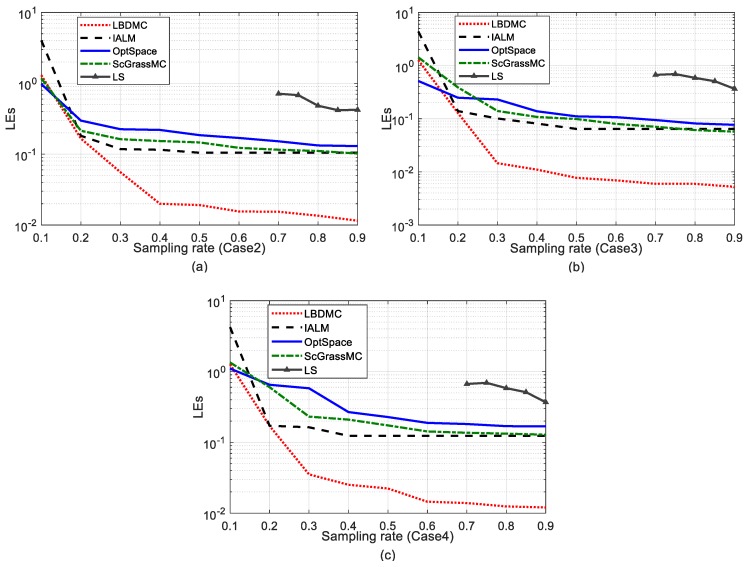
Mean localization error versus different sampling rates. The sampling rate varies successively from 10% to 90%. (**a**) Mean localization errors under Case 2: (**b**) Mean localization error under Case 3; (**c**) Mean localization error under Case 4.

**Figure 6:**
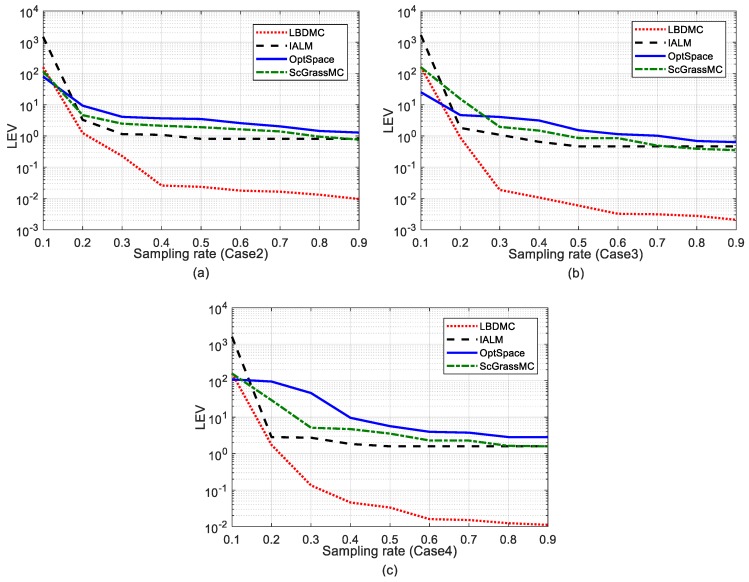
Localization error variance versus different sampling rates. The sampling rate varies successively from 10% to 90%: (**a**) Error variance under Case 2; (**b**) Error variance under Case 3; (**c**) Error variance under Case 4.

Furthermore, Figure 5 and Figure 6 display the effect of the sampling rate on the mean localization error and its variance, respectively. The comparison between the MC-based methods and Least Squares (LS), a standard localization algorithm, is shown in Figure 5. To ensure that each unknown node has sufficient available distance information in LS, that is each node has the distance measures with three or more beacons, the sampling rate was set to above 70%. It can be observed that the performance of LS was inferior to the other MC-based algorithms. Comparing Figure 5 and Figure 6, the localization error and its variance were consistent with the change of the EDM recovery errors. The LBDMC performed with lower overall errors and error variance than other methods when the sampling rate was above 15%. It is worth noting that when the sampling rate was above 30%, the mean localization error and error variance of the LBDMC were about ten times smaller than those of the other algorithms.

#### 5.2.4. Comparison of the Localization Error Cumulative Distributions

**Figure 7:**
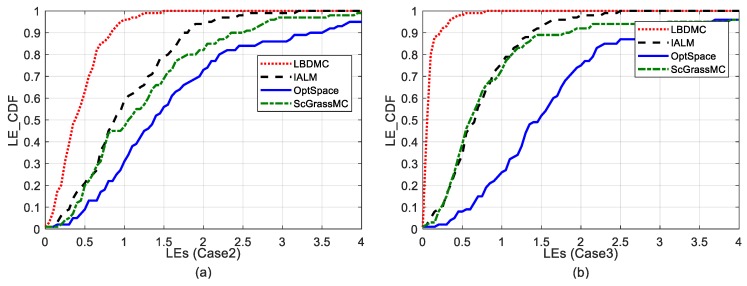
Comparison of localization error cumulative distributions. The sampling rate is fixed at 30%: (**a**) Localization errors cumulative distribution under Case 2; (**b**) Localization error cumulative distribution under Case 3.

Figure 7 depicts the localization error cumulative distributions under outlier noise and pulse noise, with the sampling rate fixed at 30%. As shown in Figure 7a, the probability of localization errors of the LBDMC being less than 1 m was up to 95%, while the counterparts of the other three algorithms were all lower than 60%. Similarly, in Figure 7b, the probability of localization errors of the LBDMC being less than 1 m was 100%, while the counterparts of IALM and ScGrassMC were lower than 80%, and the counterpart of OptSpace were only about 25%. Therefore, our proposed LBDMC had an outstanding performance.

#### 5.2.5. Comparison of Performance with Different Noise Levels

**Figure 8:**
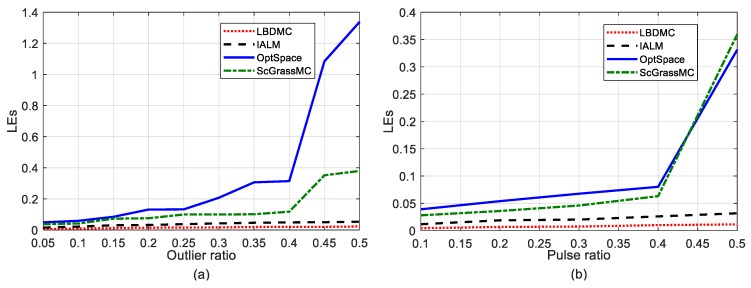
Mean localization error versus different noise levels. The sampling rate is fixed at 50%. The outlier ratio varies successively from 5 to 50%. The pulse ratio varies successively from 10 to 50%. The sampling rate is fixed at 50%: (**a**) Mean localization error versus different outlier ratios; (**b**) Mean localization error versus different pulse ratios.

**Figure 9:**
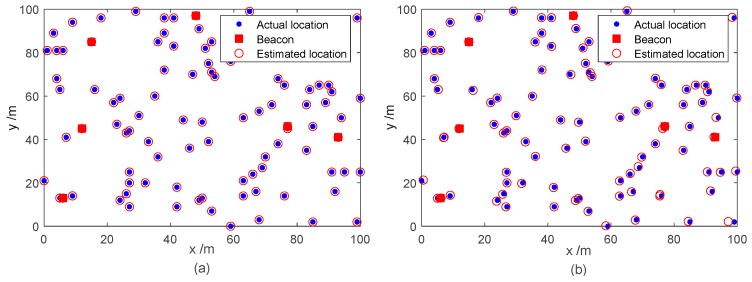
Localization results of the LBDMC with different noise environments. The sampling rate is fixed at 30%: (**a**) Localization results under Case 1; (**b**) Localization results under Case 4.

Here, we investigated the effect of different noise levels on the mean localization error. The outlier ratio varied successively from 5 to 50%, while the pulse ratio varied successively from 10 to 50%. The sampling rate was fixed at 50%. The mean localization error versus different outlier ratios and pulse ratios were evaluated, as depicted in Figure 8a,b, respectively. The performance of each algorithm deteriorated with the gradual increase of the noise ratio. OptSpace and ScGrassMC did not work well as the noise ratio increases, while the LBDMC and IALM were robust with different noise levels. Furthermore, compared with the IALM, our proposed LBDMC was more stable and achieved a smaller number of localization errors even as the noise ratio reached 50%. In addition, Figure 9a,b show the localization results of the LBDMC under Case 1 and Case 2, respectively, verifying the efficiency of the LBDMC.

## 6. Conclusions

To actualize the WSN localization from a data-missing and noisy EDM, a novel WSN localization algorithm via matrix completion based on Bregman divergence (LBDMC) was proposed in this paper. The algorithm was divided into two stages. In the first stage, the problem of EDM recovery was formulated as a matrix completion problem and the EDM estimator was obtained based on the BDMC. Then, based on the MDS method, node positioning was implemented. By comparing with IALM, OptSpace and ScGrassMC, it could be observed that the LBDMC was superior to the other three algorithms in positioning accuracy and robustness, while ensuring high efficiency under different noise conditions. Notably, when the sampling rate reached a certain extent (as an example, >30%) the mean localization error of the proposed LBDMC was about ten times smaller than that of the other three algorithms. However, under noise-free conditions, the localization accuracy of the LBDMC algorithm was not satisfactory and the convergence speed of the algorithm needed to be further improved compared to its counterpart in ScGrassMC. In addition, our proposed LBDMC is a centralized approach and future work will focus on a distributed version to reduce the limitations of computational efficiency and storage scale.
